# Effect of applied cadence in repeated sprint cycling on muscle characteristics

**DOI:** 10.1007/s00421-023-05393-z

**Published:** 2024-01-04

**Authors:** Sebastian Klich, Kamil Michalik, Bogdan Pietraszewski, Ernst A. Hansen, Pascal Madeleine, Adam Kawczyński

**Affiliations:** 1 Department of Paralympic Sport, Wrocław University of Health and Sport Sciences, 51-612 Wrocław, Poland; 2 Department of Human Motor Skills, Wrocław University of Health and Sport Sciences, 51-612 Wroclaw, Poland; 3 Department of Biomechanics, Wrocław University of Health and Sport Sciences, 51-612 Wroclaw, Poland; 4 Centre for Health and Rehabilitation, University College Absalon, 4200 Slagelse, Denmark; 5 Department of Health Science and Technology, Aalborg University, ExerciseTech, 9260 Gistrup, Denmark; 6 Department of Biomechanics and Sport Engineering, Gdansk University of Physical Education and Sport, 80-336 Gdansk, Poland

**Keywords:** Ultrasonography, Myotonometry, Sprint performance, Muscle–tendon unit

## Abstract

**Purpose:**

This study aimed to investigate physiological responses, muscle–tendon unit properties of the quadriceps muscle, and mechanical performance after repeated sprint cycling at optimal and 70% of optimal cadence.

**Methods:**

Twenty recreational cyclists performed as first sprint performance cycling test and during subsequent sessions two repeated sprint cycling protocols at optimal and 70% of optimal cadence, in random order. The muscle–tendon unit outcome measures on the dominant leg included muscle thickness, fascicle length (*L*_*f*_), pennation angle (*θ*_*p*_), and stiffness for the rectus femoris (RF), vastus lateralis (VL), and vastus medialis muscle (VM) at baseline, immediately after repeated sprint cycling, and 1-h post-exercise.

**Results:**

The results showed an increase in muscle thickness and *θ*_*p*_ in RF, VL, and VM for both cadences from baseline to immediately after exercise. The *L*_*f*_ decreased in RF (both cadences), while stiffness decreased in RF, VL, and VM at optimal cadence, and in VL at 70% of optimal cadence from baseline to immediately after exercise.

**Conclusion:**

The present study revealed that the alterations in muscle characteristics were more marked after repeated sprint cycling at optimal cadence compared with a lower cadence most likely as a result of higher load on the muscle–tendon unit at optimal cadence.

**Supplementary Information:**

The online version contains supplementary material available at 10.1007/s00421-023-05393-z.

## Introduction

Cyclists use different cadences based on field conditions or gear combinations. A cadence of ~ 90 RPM is considered optimal for high level of performance in professional cyclists, but lower cadences (< 80 RPM) are commonly chosen for training, especially to improve endurance performance (Hansen and Rønnestad, 2017). Abbiss et al. (2009) suggest that low cadence is observed by endurance cyclists during uphill mountain ascents. It shows that training at low cadence provides significant effect on cyclist’s performance related to increased aerobic capacity (Paton et al. 2009). Also, Harnish et al. (2007) have reported that lower cadences are preferred in standing position for exercises at 50, 65, and 75% of peak power output (57, 62, and 66 RPM, respectively) compared with the seated position. Training at low cadences is often performed to improve cyclists’ strength and is found to be more effective in developing sprint performance than high cadences (110–120 RPM) (Paton et al. 2009). Moreover, training sessions at a relatively low cadence may result in specific training adaptations of muscles. Thus, the understanding of acute changes in single-joint isokinetic maximal voluntary contractions and muscle–tendon morphological properties can contribute to understand long-term adaptations due to regular training (Kordi et al. 2017, 2020).

The term “optimal” cadence has been used widely in the literature; however, its understanding is not straightforward. The cadence might be considered as optimal when the pedaling rate results in power peak output (PPO), as e.g., in a sprint (Dorel et al. 2005; Samozino et al. 2007). Optimal cadences have been reported in previous studies ranging from 120 to 140 RPM among elite cyclists (Abbiss et al. 2009; Ansley and Cangley 2009; Hodson-Tole et al. 2020; Samozino et al. 2007; Kordi et al. 2020), 122 RPM in healthy males (Hansen et al. 2002), and ~ 110 RPM in recreational cyclists (Taylor-Haas et al. 2022). Short-term sprint cycling performance at high cadence may lead to decrease in muscle force due to fatigue development (Abbiss et al. 2009; Sarre and Lepers 2005). Still, there is to date no studies, which have thoroughly investigated the role of cadence during repeated sprint cycling. The PPO generated by cyclists depends also on intrinsic factors, such as muscle morphology (distribution of fiber types, muscle size and volume), and mechanical properties (Hill’s force–velocity relationship) (Martin et al. 2007; Samozino et al. 2007; Hansen et al. 2002). Previous studies have investigated physiological determinants of PPO based on different morphological properties such as lean thigh volume (Dorel et al. 2005), fat-free mass (Duché et al. 2002), and isometric quadriceps strength (Driss et al. 2002). Kordi et al. (2017) have reported strong correlations between PPO and peak isometric force of knee extensors, while moderate correlations have been shown with hip extensor and knee flexor force. Additionally, these authors reported strong correlations between PPO and both quadriceps and hamstrings muscle volume, as well as pennation angle of the vastus lateralis muscle during a sprint cycling performance test. It has also been suggested that other morphological properties, such as muscle thickness might be a determinant of PPO (Kordi et al. 2020). In line with that, the thickness of the vastus lateralis muscle has been shown to be correlated to isometric and dynamic strength (Secomb et al. 2015). Moreover, muscle architecture, i.e., fascicle length and pennation angle, is known as an important predictor of PPO in sprint performance (Kordi et al. 2019, 2020; Kumagai et al. 2000).

In a biomechanical context, muscle stiffness refers to the resistance of tissue during passive stretching which depends on the type of external force applied and the deformation of the structure caused by this force (Baumgart 2000). Increased level of stiffness has been related to a higher risk of strain and stress injuries (Pruyn et al. 2012). Assessing the biomechanical properties of the musculoskeletal system is challenging because striated muscle is an anisotropic and viscoelastic complex composed of both active and passive structures (Gennisson et al. 2010). Concerning changes in tendon thickness, both increases and decreases have been reported immediately after mechanical loading (e.g., Obst et al. 2013; Tardioli et al. 2012; Shalabi et al. 2004). The current body of literature underlines a variety of approaches when investigating physiological predictors and determinants of PPO, but does not consider recent advances in the evaluation of morphological properties such as muscle stiffness. Previous studies have analyzed fascicle lengths of vastus lateralis (VL) at different cadences in recreational cyclists (Austin and Nilwik, 2010; Brennan et al. 2018,2019). Austin and Nilwik, (2010) have used an experimental procedure with cadences at 50 and 80 RPM and at power outputs of 100 and 250 W. The findings from this study have demonstrated that a decrease in fascicle length at given pedaling rate (for 50 and 80 RPM) may reduce power production. Brennan et al. (2018, 2019) have found that fascicle shortening increases when cadence decreases (40–60–80–100 RPM) at 2.5 W⋅kg^−1^. Concerning pennation angle, Kordi et al. (2020) have reported greater pennation angle of VL in sprinters compared with endurance cyclists. To date, no studies have used a repeated sprint exercise procedure at optimal and suboptimal cadence to investigate muscle–tendon unit morphological and mechanical properties, as well as architecture. In the present study, we investigated physiological responses, muscle–tendon unit properties of the quadriceps muscle, and mechanical performance after repeated sprint cycling at (i) optimal and (ii) 70% of optimal cadence among recreational cyclists. We hypothesized that repeated sprint cycling will result in fatigue-related changes delineated by changes in muscle/tendon thickness and architecture, as well as decrease in muscle stiffness. Moreover, we hypothesized that repeated sprint cycling exercises at optimal cadence compared with 70% of optimal cadence will cause greater changes related to fatigue in muscle–tendon properties.

## Materials and methods

### Study overview

**Fig. 1:**
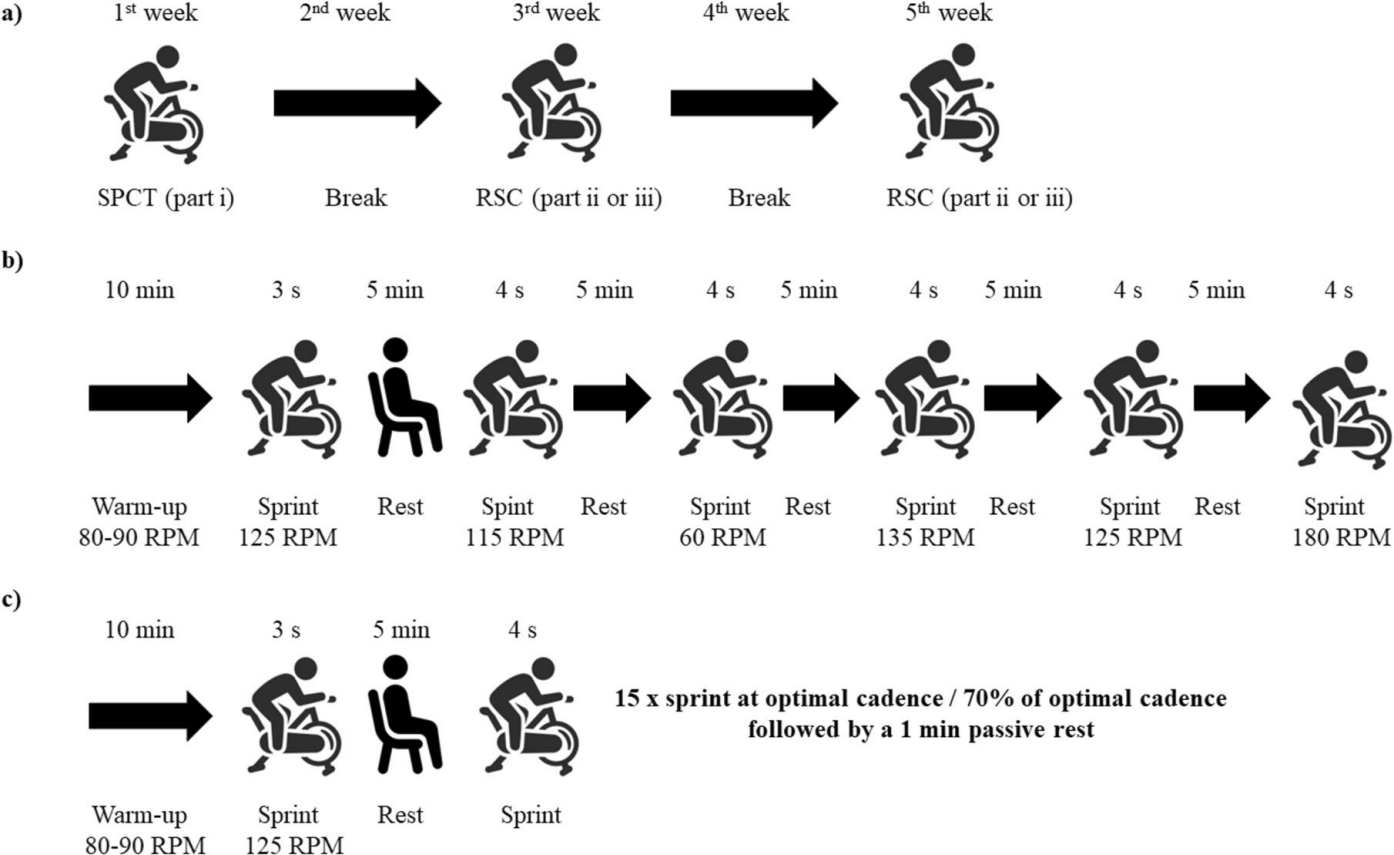
The total duration of the experiment was 5 weeks (**a**). The sprint cycling performance test is illustrated in (**b**). The repeated sprint cycling sessions are illustrated in (**c**)

This study design was a cross-sectional study with repeated measures, where (1) mechanical performance, (2) morphological, (3) mechanical alterations, and (4) architecture in the quadriceps muscle–tendon unit properties at three time points (at baseline, immediately after, and 1 h after repeated sprint cycling) were investigated, similarly to Cipryan et al. (2017), but with a shorter post-exercise recovery period. All measurements were made on the dominant side of the lower extremity, by the same skilled experimenter. The study consisted of three laboratory sessions separated by 1 week: (*i*) sprint performance cycling test (SPCT), (*ii*) repeated sprint cycling (RSC) at optimal cadence, and (*iii*) RSC at 70% of optimal cadence (Fig. 1). Note that parts (*ii*) and (*iii*) were conducted in a randomized balanced order.

### Participants

Twenty recreational and amateur cyclists, aged 18–35 years, participated. Characteristics, including information on the participants’ training, are presented in Table 1. All participants had experience in sprint and peak power training sessions (e.g., on a velodrome and/or cycle ergometer). All cyclists were tested to identify the dominant leg during pedaling, in agreement with previously published procedures (Klich et al. 2020). The inclusion criteria were: (1) total cycling distance performed in a year > 5,000 km, and (2) training experience > 5 years. The exclusion criteria included: (1) current or previous thigh and knee injury or pain symptoms and (2) prior history of surgery in the lower extremity. Each subject read and signed an informed consent form approved by the Senate Research Ethics Committee (project identification code: 1/2019 approval date: 11.01.2019). The study was conducted according to the Declaration of Helsinki.

**Table 1:** Mean ± SD of the participant characteristics

Variables	Recreational cyclists *n* = 20
Age (year)	26.6 ± 6.2
Sex	♂: 20
Body height (m)	1.78 ± 0.6
Body mass (kg)	78.2 ± 8.7
Body Mass Index (kg/m^2^)	24.6 ± 1.2
Lower extremity dominance:	
Right	16
Left	4
Training experience (years)	7 ± 5
Total cycling distance performed in the preceding year (km)	6,625 ± 2,400
Optimal cadence (RPM)	108 ± 7
70% of optimal cadence (RPM)	76 ± 5

The G*Power software (version 3.1.9.2; Kiel University, Kiel, Germany) was used to estimate the required sample size. A sample size estimated with a repeated measure ANOVA within and between factors, an α of 0.05, set a minimum expected effect size (Cohen’s *f*) of 0.4, β of 0.90 and correlation among repeated measures of 0.5. The procedure included a minimum number of 18 participants, but 2 extra participants were recruited to account for potential dropout.

### Experimental procedure

Sprint cycling performance test (SPCT, i) and repeated sprint cycling sessions (RSC, ii and iii) were performed on a cycle ergometer (Excalibur Sport, Lode BV, The Netherlands). During the first week of data collection, all cyclists performed SCPT, while during the next 2 weeks, RSC was performed at optimal and at 70% of optimal cadence (in random order) (Fig. 1a–c).

The SCPT was performed to determine key mechanical determinants of cycling performance established by power–cadence and torque–cadence relationships using a quadratic and linear equation (Dorel et al. 2005; Gardner et al. 2007). To investigate power–cadence relationships, the apex of the relationship was found by interpolation, to reflect *PPO*. Afterward, *PPO* was calculated relative to body mass [W/Kg] and cadence at PPO (*C*_*OPT*_) was extracted. Finally, we extrapolated the torque–cadence relationships, maximal torque (*τ*_*max*_), and maximal cadence (*C*_*MAX*_) in line with Kordi et al. (2020). The test was initiated by a 10 min standardized warm-up at 80–90 RPM at a submaximal intensity of 100–150 W, followed by a maximal 3-s sprint at 125 RPM. After a 5-min passive rest, the participant performed the test. The SCPT consisted of five iso-velocity all-out 4-s sprints at five different cadences selected in the same order, i.e., 115, 60, 135, 125, and 180 RPM interspaced by 5 min passive rest (Fig. 1b).

The warm-up was like that in part *i*. The order in which participants performed RSC at individual optimal or at 70% of optimal cadence was randomly balanced using Research Randomizer v.4. Both sessions included 15 repetitions of the 4-s all-out iso-velocity sprint. These sprints were separated by 60 s passive rest, sitting on the ergometer cycle (Fig. 1c).

### Morphological properties and muscle architecture

Morphological properties of the quadriceps muscle–tendon unit were investigated using ultrasonography (Alpinion X-CUBE 90, Alpinion, South Korea) with a 10.0 MHz and 60 mm linear array transducer (Single Crystal, Alpinion, South Korea) in grayscale B-mode. A skilled examiner (7 years of experience) obtained ultrasound images of the rectus femoris (RF), vastus lateralis (VL), and vastus medialis muscle (VM).

**Fig. 2:**
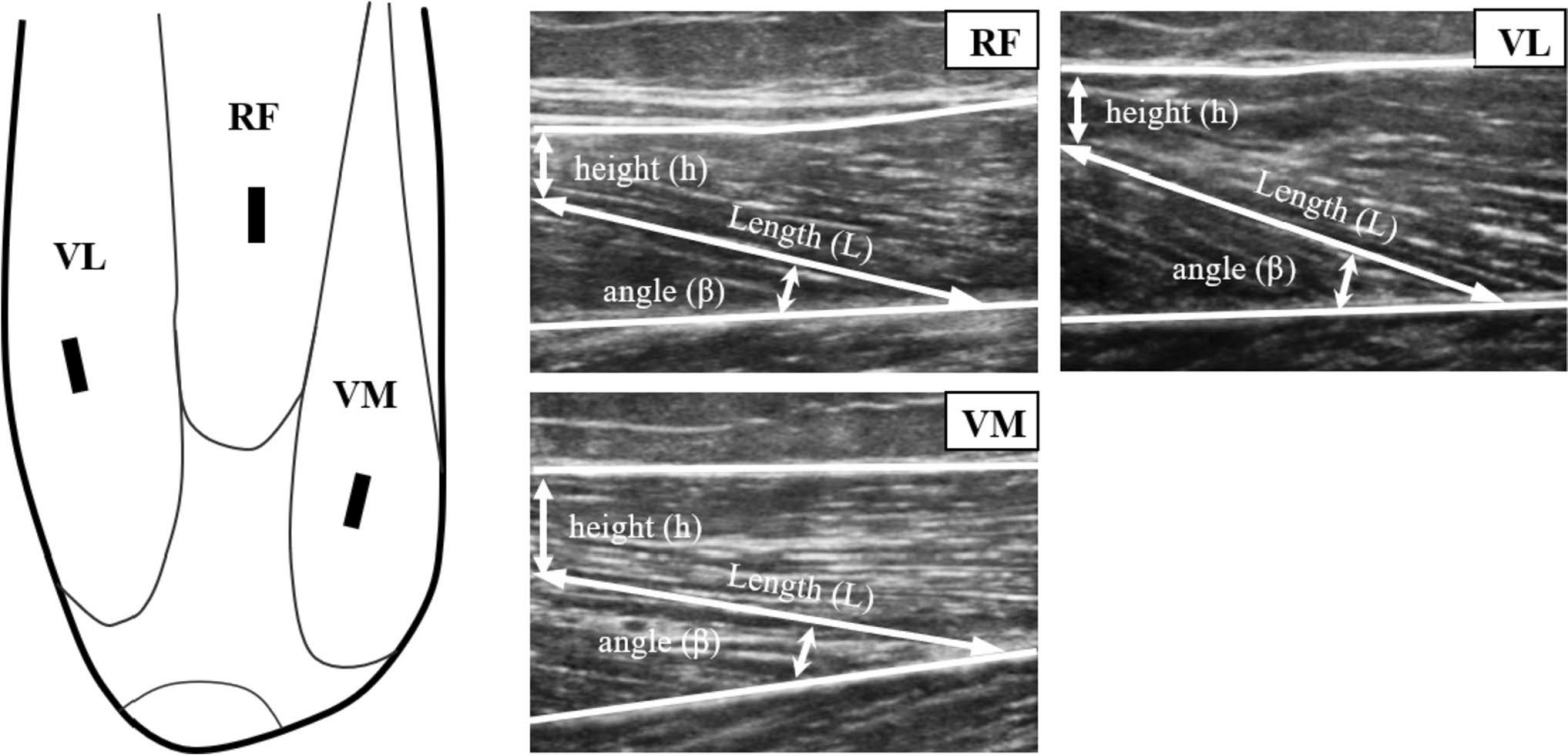
Evaluation of fascicle length of the quadriceps muscle. Reference points and locations including for rectus femoris (RF), vastus lateralis (VL), and vastus medialis (VM). The transducer was placed at 56%, 39%, and 22% of total thigh length, respectively (Blazevich et al. 2006). In this figure “L” indicates observable fascicle length, *h* indicates the perpendicular distance between the superficial aponeurosis and distal point of the fascicle, and *β* indicates the angle between the fascicle and the superficial aponeurosis

Before the ultrasonographic examination began, the examiner measured the total length of the thigh with a ruler from the superior aspect of the patella to the anterior superior iliac spine. Next, all five reference points for each muscle and tendon were marked on the skin with a permanent marker to maintain consistency within a day session. For ultrasound projections of each muscle, the volunteers were lying in a supine position with their dominant knee flexed at approximately 45° (Blazevich et al. 2006). A pillow was placed under the popliteal space during the evaluation. For RF, VL, and VM, the ultrasonographic probe was oriented in the longitudinal axis (thickness, *L*_*f*_, and pennation angle, *θ*_*p*_*)* of the quadriceps muscle. For RF, the ultrasound probe was placed on the medial aspect of the thigh at 56% of the thigh length. For VL, the transducer was positioned at 39% of the thigh length, while it was at 22% of the thigh length for VM. The transducer was placed in the longitudinal axis to the thigh muscles (Blazevich et al. 2006). The exact position of the probe was considered when several fascicles were defined (Engelina et al. 2014) (Fig. 2).

Thickness was measured as the distance between deep and superficial aponeuroses, while *θ*_*p*_ was determined as an angle between a fascicle and the deep aponeurosis (Ema et al. 2013). Moreover, the fascicle length (*L*_*f*_) was calculated from Eq. (1):

1 $${L}_{f}=L+(h\div {\text{sin}}\left(\beta \right),$$

where *L* was the observable fascicle length, *h* was the perpendicular distance between the superficial aponeurosis and distal point of the fascicle, and *β* was the angle between the fascicle and the superficial aponeurosis.

The quadriceps tendon (QT) was defined proximal to the upper edge of the patella. For the patellar tendon (PT), the ultrasound probe was positioned distal to the patella. The measurement procedures followed previous procedures by Klich et al. (2020) including three reference points, i.e., at 5, 10, and 15 mm from the most hyperechogenic point on the patella. The thickness of the PT was evaluated from four reference points, i.e., at 5–10 to 15–20 mm inferior to the apex of the patella. All measurements were averaged to a single value.

### Mechanical properties

Muscle–tendon unit stiffness was assessed using a myotonometer (MyotonPro, Myoton Ltd., Estonia). The position for this examination was identical with the position applied for ultrasonographic imaging, i.e., the volunteers were lying in a supine position with their dominant knee flexed at approximately 30° supported by a pillow under the popliteal space. Stiffness of the quadriceps muscle was assessed on the dominant lower extremity over seven reference points, including muscles: rectus femoris (RF)—musculotendinous points located distal from the initial attachment and terminal attachment; vastus lateralis (VL)—three points on the muscle belly in three equal parts; vastus medialis (VM)—middle point of the muscle belly, as well as tendons: quadriceps tendon (QT)—the 1/3 proximal between the upper edge of patella and patellar tendon (PT)—a midway point between the patella distal and the tuberosity of tibial (knee flexed at 90°) (Klich et al. 2020). All reference points were marked on the skin with a permanent marker to maintain consistency between testing days. The rater placed the Myoton Pro probe perpendicular to the tested area. Next, the probe generated three impulses exerted on the testing area. Measurements were taken at baseline, immediately after, and 1 h after sprint performance cycling test. The relative reliability was excellent for stiffness of VL muscles (ICC_2, 1_ 0.89). The absolute reliability showed that SEM was 14 N/m, while MDC_90%_ was 36 N/m in a pilot study with ten participants.

### Statistical analysis

The SPSS statistical software (version 18., SPSS Inc., Chicago, Illinois, USA) was used for data analysis. Mean values ± standard deviation (SD) or confidence interval (CI 95%) are reported. The normality of the data distribution was tested using Shapiro–Wilk tests, while homogeneity of variance was analyzed by Levene’s test. The analyzed data were normally distributed for all parameters, while the variances were equal for all parameters. A paired samples *t* test was used to evaluate differences between both sessions (at optimal cadence and at 70% of optimal cadence) for mechanical performance (*PPO*, average *PPO*, *τ*_*max,*_ and average *τ*). The effect size was estimated using Cohan’s d (d), classified as very small (0.01), small (0.2), medium (0.5), large (0.8), very large (1.2), and huge (2.0) (Sawilowsky 2009). Finally, a two-way RM-ANOVA with *Time* (baseline, immediately post-exercise, and 1 h post-exercise) and *Cadence* (optimal and 70% of optimal) was conducted for morphological, mechanical properties, and architecture (thickness, stiffness, *L*_*f*_,* θ*_*p*_) in quadriceps muscles (RF-VL-VM) and tendons (QT-PT). If a significant interaction between factors was found, the Bonferroni adjustment for multiple comparisons was used for post hoc tests (*p* = 0.02). The effect size was estimated using partial eta square (η^2^), classified as small (0.2 < η^2^ < 0.049), medium (0.5 < η^2^ < 0.79), or large (η^2^ ≤ 0.8) (Richardson 2011). For all statistical tests beside post-hoc tests, a *p*-value ≤ 0.05 was considered significant.

## Results

### Mechanical performance

Table 2 reports the mean ± SD of mechanical performance. The paired samples *t *test showed that cyclists reached greater *PPO* during repeated sprint cycling at optimal cadence (1,224 ± 189 W) compared with sprint cycling at 70% of optimal cadence (1,082 ± 164 W) (*t* = 2.5; *p* = 0.018; *d* = 0.80). The optimal cadence resulted also in significantly lower *τ*_*max*_ and *τ*_*AV*_ (109 ± 14 Nm and 91 ± 14 Nm, respectively) compared with 70% of optimal cadence (137 ± 17 Nm and 125 ± 16 Nm, respectively) (*t* = − 5.5; *p* ≤ 0.001; *d* = 1.80 and *t* = − 7.2; *p* ≤ 0.001; *d* = 2.26 for both, respectively).

**Table 2:** Physiological response and mechanical performance measurements after post warm-up and immediately post-exercise repeated sprint cycling (RSC) at optimal and at 70% of optimal cadence. Mean ± SD values

	RSC with optimal cadence	RSC with 70% optimal cadence	Δ (OPT – 70% OPT) (95% CI)	*p* value
Mechanical performance				
PPO [W]	1,224 ± 189	1,082 ± 164	142 (103,180)	*p* = 0.018
PPO_AV_ [W]	1,015 ± 176	980 ± 150	35 (32,68)	*p* = 0.065
τmax [Nm]	109 ± 14	137 ± 17	− 28 (− 33,− 23)	*p* < 0.001
τ_AV_ [Nm]	91 ± 14	125 ± 16	− 34 (− 37,− 31)	*p* < 0.001

### Morphological properties

**Fig. 3:**
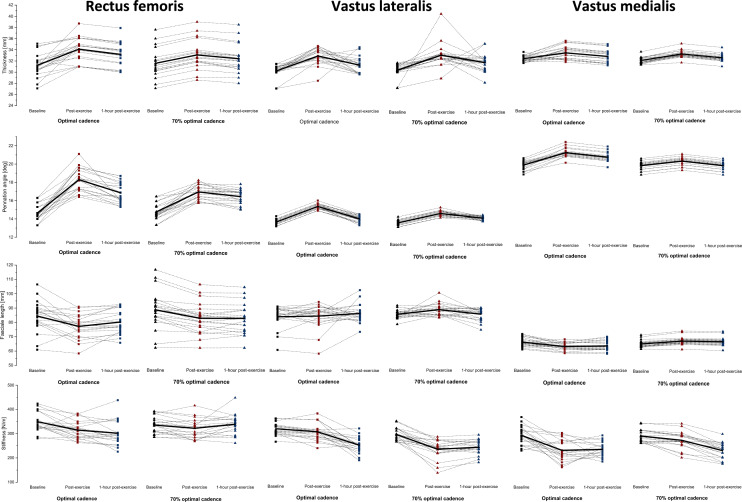
Mean ± SD of the thickness (mm), stiffness (N/m), pennation angle (o), and fascicle length (mm) in the dominant lower extremity at an optimal cadence (OPT CAD) and 70% of optimal cadence (70% OPT CAD). Measurements were performed for the following muscles: rectus femoris, vastus lateralis, and vastus medialis. OPT CAD optimal cadence (black line); 70% OPT CAD 70% of optimal cadence (red line)

**Fig. 4:**
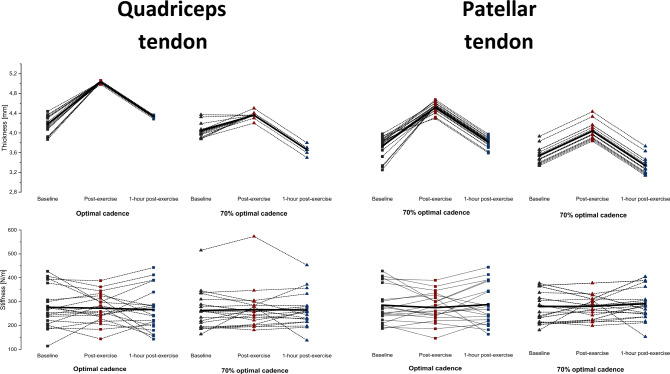
Mean ± SD of the thickness (mm), stiffness (N/m), pennation angle (o), and fascicle length (mm) in the dominant lower extremity at an optimal cadence (OPT CAD) and 70% of optimal cadence (70% OPT CAD). Measurements were performed for the quadriceps and patellar tendon. OPT CAD optimal cadence (black line); 70% OPT CAD 70% of optimal cadence (red line)

Figures 3 and 4 show the mean ± SD of the thigh muscle–tendon unit morphological properties at baseline, immediately post-exercise, and at 1-h post-exercise after both sprint cycling protocols. The two-way RM-ANOVA revealed a statistically significant main effect of *Time* (*F*_2,236_ = 87.4, *p* ≤ 0.001, η^2^ = 0.43) for quadriceps muscles. Moreover, a statistically significant main effect of *Time* (*F*_1,78_ = 19.0, *p* ≤ 0.001, η^2^ = 0.20) and *Cadence* (*F*_1,78_ = 65.3, *p* ≤ 0.001, η^2^ = 0.46) was showed for quadriceps tendons. Finally, a statistically significant interaction effect of *Time* × *Cadence* (*F*_1,78_ = 104.9, *p* ≤ 0.001, η^2^ = 0.57) was found for quadriceps tendons. Muscle thickness increased significantly from baseline to immediately post-exercise in RF, VL, and VM (*p* ≤ 0.001 for all muscles) at both optimal and 70% of optimal cadence from baseline to immediately post-exercise, as well as decreased significantly in VL (*p* ≤ 0.01 for both cadences) from immediately post-exercise to 1-h post-exercise. A significant decrease in RF was found only immediately post-exercise at optimal cadence (*p* ≤ 0.001). The QT and PT thickness increased significantly from baseline to immediately post-exercise (*p* ≤ 0.001 for both) and decreased from immediately to 1-h post-exercise (*p* ≤ 0.001 for both) at both optimal and 70% of optimal cadence. Moreover, the QT and PT thickness was significantly thicker immediately (*p* ≤ 0.001 for both) and lower 1-h post-exercise (*p* ≤ 0.001 for both) at optimal cadence compared with 70% of optimal cadence.

### Muscle architecture

Muscle architecture was established *θ*_*p*_ and *L*_*f*_ (mean ± SD) of the thigh muscles at baseline, immediately post-exercise, and 1-h post-exercise after both sprint cycling protocols. The two-way RM-ANOVA revealed a statistically significant main effect of *Time* (*F*_2,236_ = 206.7, *p* ≤ 0.001, η^2^ = 0.64) for *θ*_*p*_. The *θ*_*p*_ increased significantly in RF, VL, and VM from baseline to immediately post-exercise (*p* ≤ 0.001) and decreased in all muscles at 1-h post-exercise compared with immediately post-exercise (*p* ≤ 0.001) at both cadences. The *L*_*f*_ decreased significantly in RF from baseline to immediately post-exercise (*p* ≤ 0.001) at both cadences. The *L*_*f*_ increased in VL from baseline to immediately post-exercise (*p* = 0.01) at 70% of optimal cadence (Fig. 3).

### Mechanical properties

Figures 3 and 4 shows the mean ± SD of the thigh muscle–tendon unit stiffness at baseline, immediately post-exercise, and 1-h post-exercise after both sprint cycling protocols. The two-way RM-ANOVA revealed a statistically significant main effect of Time (F_2,236_ = 7.3, *p* = 0.001, η^2^ = 0.06) for quadriceps muscles stiffness. Stiffness decreased significantly in RF, VL, and VM (*p* ≤ 0.001 for all) from baseline to immediately post-exercise while it increased significantly in VL (*p* = 0.008) from baseline to 1-h post-exercise at optimal cadence. At 70% of optimal cadence, stiffness decreased significantly in VL (*p* ≤ 0.001) from baseline to immediately post-exercise, and in VM (*p* = 0.02) from baseline to 1-h post-exercise. Furthermore, significant differences between time points were found for QT and PT at optimal and 70% of optimal cadence (*p* = 0.02).

## Discussion

Understanding repeated sprint performance at different cadences might be useful to explain the mechanisms undergoing during fatigue. This study investigated for the first-time post-exercise alterations in muscle–tendon unit characteristics, expressed by morphological and mechanical properties, as well as muscle architecture of the quadriceps muscle after repeated sprint cycling protocol at both optimal and 70% of optimal cadence. Previous studies have not studied changes in muscle–tendon unit properties and fatigue-induced mechanisms after repeated sprint performance exercises. Only the changes in muscle volume, thickness, *θ*_*p*_, and *L*_*f*_ have been analyzed between groups, e.g., sprint and endurance cyclists (Kordi et al. 2020; Lee et al. 2021). The findings of this study supported the hypotheses and resulted in suggestions regarding the physiology behind acute fatigue-induced changes in the quadriceps muscle including the patellar tendon. Thus, the current study provides novel information concerning alteration in the muscle–tendon unit properties. We observed a greater thickness, *θ*_*p*_, and *L*_*f*_, compared with Lee et al. (2021) mostly due to a difference in the subject's characteristics, i.e., training status.

### Mechanical performance

Muscle–tendon unit properties of the quadriceps muscle are an important indicator of sprint cycling performance. The results of our study demonstrated differences between both sprint cycling sessions, presenting greater average PPO and lower average torque at optimal cadence compared with 70% of optimal cadence. In contrast to previous studies that focused on professional’s cyclists, we examined recreational endurance cyclists with a PPO of 1,224 ± 189 W (optimal cadence) and 1,082 ± 164 W (70% of optimal cadence). These results are similar to those reported for elite endurance cyclists (1,122 ± 65 W) (Calbet et al. 2003), and higher as in trained (942 ± 136 W) (Kordi et al. 2019), recreational (941 ± 124 W) (Leong et al. 2014), and college cyclists (908 ± 100 W) (Lee et al. 2021).

### Morphological, mechanical properties, and architecture of the quadriceps muscle

Ultrasound imaging has been used over the preceding two decades to evaluate the quadriceps muscle and tendons alterations after different fatigue-induced protocols, including laboratory and field-based environments (Klich et al. 2023, 2020; Bouillard et al. 2014; Brancaccio et al. 2008), and has shown excellent reliability (Blazevich et al. 2006).

Brancaccio et al. (2008) performed an incremental cycling protocol at a cadence of 60–70 RPM and reported a significant increase of 8% in RF muscle thickness. In our study, we used both a similar cadence (76 RPM) at 70% of the optimal cadence and at optimal cadence (108 RPM). We observed a larger thickness of RF at immediately post-exercise at optimal cadence compared with 70% of optimal cadence. This could be explained by a greater vascular perfusion for glucose combined with a micro-inflammatory process (Kayala et al. 2012). Furthermore, a larger increase in *θ*_*p*_ and a simultaneous decrease in *L*_*f*_ at optimal cadence were also found. According to Kawakami et al. (1993), an increase in muscle thickness may cause an increase in *θ*_*p*_ by enlarging the muscle volume. The increases in thickness and, therefore, *θ*_*p*_ could be associated with a geometrical rearrangement due to the higher water and fluids (e.g., blood or inflammatory substances) contents. Indeed, an increase in the amount of liquid inside a given volume may increase muscle thickness and pennation angle and, consequently, reduce fascicle length, as reported in the current study. A larger increase in RF thickness and *θ*_*p*_ at optimal cadence can also be related to a higher muscle load (Ansley and Cangley 2009). Our results are in line with, Csapo et al. (2011), who reported acute changes in muscle architecture in response to resistance exercise, delineated by an increase in vastus lateralis muscle thickness and *θ*_*p*_, with a simultaneous decrease in *L*_*f*_ as a possible result of an additional decrease in tendon stiffness due to shortening of the sarcomere. The increase in *L*_*f*_ immediately post-exercise (at 70% of optimal cadence) could also be explained by an increase in thickness, while *θ*_*p*_, and *L*_*f*_ may indicate muscle potential in force-generating capacity, especially for VL during cycling (Bieuzen et al. 2007). Finally, the recovery analysis showed a significant decrease in the thickness of RF and VL, and in *θ*_*p*_ in all muscles after 1-h post-exercise at both cadences. Csapo et al. (2011) have reported a different recovery time for morphological properties and muscle architecture, e.g., muscle thickness: 30 min, *θ*_*p*_: 15 min, and *L*_*f*_: 5 min recovery time.

In our study, we re-examined cyclists after 1 h and thickness and *θ*_*p*_ had not returned to baseline values. The reason for different recovery could be explained by different muscle load (Csapo et al. 2011). Still, our results showed that repetitive sprints did not enable full recovery 1-h post-exercise of morphological properties and architecture of the quadriceps muscles regardless of the cycling cadence underlining risk of overuse (Klich et al. 2023). In summary, we observed greater alterations in morphological properties and muscle architecture, especially in VL at optimal cadence compared with 70% of optimal cadence. The more marked changes in muscle properties could be related to hypervascularity due to higher level of muscle activity at higher cadences (Bieuzen et al. 2007).

We also found a decrease in RF, VL, and VM stiffness (from 10 to 19%) at optimal cadence, and in VL stiffness (18%) at 70% of optimal cadence. There was also an increase in VL stiffness (16%) at 1-h post-exercise at optimal and at 70% of the optimal cadence. Overall, the quadriceps muscle thickness and stiffness might be influenced by the level of cadence during repeated sprint performance. Sprint exercises at higher cadences (in our study optimal cadence at 108 ± 7 RPM) typically involve fast-twitch muscle fibers to produce greater power. Fast-twitch fibers are characterized by greater twitch tensions, and faster conduction velocities compared with slow-twitch fibers (Mendez-Villanueva et al. 2008). An immediate post-exercise increase in muscles thickness could be explained as the result of muscle loading causing fluid accumulation resulting in a decrease of muscle stiffness (Brancaccio et al. 2008).

### Quadriceps and patellar tendon properties

There was also a significant increase in QT and PT thickness immediately after the sprint cycling protocol at an optimal cadence contrary to the findings reported in previous review papers (Obst et al. 2013; Tardioli et al. 2012). We have previously reported an increase in these tendons after fatigue-induced protocol (Klich et al. 2023), and after individual pursuit races (Klich et al. 2020). This observation could be explained by the high-intensity loads leading to tendons thickening (Fisker et al. 2017). Moreover, previous studies have also reported increases on tendon thickness (Hannafin and Arnoczky 1994; Svensson et al., 1985; Shalabi et al. 2004) that may be related to an increase in water content and interstitial fluid movement (Wearing et al. 2013). Another potential explanation is to the paratenon and its exercise-induced alterations due to thickening. It should be noted that the paratenon might not be defined on ultrasound images and post-exercise measurement could also include this layer of connective tissue (Paulos et al. 1983). Still, these findings can be contra intuitive as tendon adaption usually requires weeks or months (Wiesinger et al. 2015). Obst et al. (2013) explained the decrease in tendon thickness occurring immediately after exercise by a greater hydrostatic pressure and fluid movement to the peritendinous space. Still, more studies are needed to clearly show and explain the changes in tendon properties immediately post-exercise.

### Practical implications

This study has some practical implications for amateur cyclists and cycling coaches. Firstly, repeated sprint cycling exercises are more popular for improving intermittent performance and to optimize training process in different sports (Samozino et al. 2007). This experimental protocol may be useful to design personalized training programs that fits with athlete’s unique performance goals. Thus, it is important to examine acute responses of muscle–tendon units after using different cadences. Secondly, these novel findings should be included in the training process, especially in the PPO and/or torque endurance improvement phase (e.g., during uphill cycling). Finally, more strenuous training regimes need longer rest periods between sessions to avoid overuse syndrome in muscle–tendon units.

### Limitations

Some limitations should be pointed out to improve future studies. First, we recruited only recreational cyclists performing endurance training. Future studies should include either sprint or endurance cyclists to compare the magnitude of alterations, as well as group-dependent differences in the quadriceps muscle. Second, only male participants were recruited, and the current findings should not be extrapolated to females. Finally, we used a geometric approximation (i.e., linear extrapolation) to estimate fascicle length. Still, this can result in error due to fascicle curvature (Franchi et al. 2020). Future studies should consider using manual linear extrapolation or extended field of view imaging.

## Conclusion

This study assessed the morphological and mechanical properties, as well as muscle architecture, following repeated sprint cycling exercises performed at both optimal cadence and 70% of optimal cadence in recreational cyclists. Ultrasonographic and myotonometric evaluation of the quadriceps muscle showed greater thickness and *θ*_*p*_ in RF, while a greater *θ*_*p*_ and stiffness in VL immediately post-exercise at optimal cadence compared with 70% of optimal cadence. Post-exercise changes are likely related to the increased muscular load on the quadriceps and the maximization of force production at optimal cadence. Moreover, the *θ*_*p*_ in RF and VM were greater 1-h post-exercise at optimal cadence compared with at 70% of optimal cadence. We suggest that repeated sprint cycling exercises at the optimal cadence might cause muscle fiber micro-damage, requiring additional time for recovery. These findings suggest that the choice of cadence may also have a significant impact on muscle characteristics that could be relevant to consider for training strategies.

### Supplementary Information

Below is the link to the electronic supplementary material.

Supplementary file1 (XLSX 39 KB)

## Data Availability

Data supporting the findings of this work are available within the article and its Supporting Information files.

## Abbreviations

CI 95%: Confidence intervals
C_MAX_: Maximal cadence
C_OPT_: Cadence at PPO
d: Cohen’s d
h: The perpendicular distance between the superficial aponeurosis and distal point of the fascicle
L_f_: Fascicle length
PPO: Peak power output
PPO_AV_: Average peak power output
PT: Patella tendon
RF: Rectus femoris muscle
RM-ANOVA: Repeated measure of analysis of variance
RPM: Revolutions per minute
RSC: Repeated sprint cycling
SD: Standard deviation
SPCT: Sprint performance cycling test
QT: Quadriceps tendon
VL: Vastus lateralis muscle
VM: Vastus medialis muscle
β: The angle between the fascicle and the superficial aponeurosis.
η^2^: Eta square
θ_p_: Pennation angle
τ_max_: Maximal voluntary torque
